# Optimizing Subcutaneous Antibody Dosing Regimens Through Operating Space Maps: rHuPH20 Case Study

**DOI:** 10.1007/s10928-026-10037-8

**Published:** 2026-06-29

**Authors:** Ryan P. Nolan, Harish Chintakuntla

**Affiliations:** Halozyme Therapeutics, 12390 El Camino Real, San Diego, CA 92130 USA

**Keywords:** Pharmacokinetics, Antibodies, Subcutaneous, Hyaluronidase, Drug delivery, Dose optimization

## Abstract

Therapeutic antibody dosing regimens are often constrained by formulation and administration factors that limit flexibility in dose and schedule. Subcutaneous (SC) delivery offers advantages over intravenous (IV) administration—such as improved convenience and potentially more favorable pharmacokinetic (PK) profiles—but is typically restricted by injection volume, requiring lower doses and more frequent dosing. Recombinant human hyaluronidase PH20 (rHuPH20) enables rapid administration of high-dose, high-volume therapeutics, thereby expanding the feasible SC delivery landscape. Despite these advances, biopharmaceutical development teams often lack tools to quantify the strategic impact of SC-enabling technologies and interpret PK simulations in a decision-ready format. This work introduces Operating Space Maps, a visual PK simulation framework that organizes multiple dosing scenarios into an intuitive landscape of dose–frequency options. Using rHuPH20 as a case study, a two-compartment PK model is applied to simulate representative antibody regimens for two illustrative scenarios: converting IV regimens to SC with rHuPH20 and extending SC dosing intervals beyond standard limits with rHuPH20. For example, relative to a 1000 mg IV benchmark regimen, a 1400 mg SC dose with rHuPH20 maintains equivalent average exposure while reducing C_max_ by approximately 50%. The methodology is broadly applicable to other SC-enabling technologies for which sufficient foundational data exist to parameterize the model. By making trade-offs between C_min_, C_avg_, and C_max_ explicit and integrating SC delivery constraints, Operating Space Maps enable cross-functional development teams to evaluate regimen feasibility, optimize target product profiles, and guide strategic decisions early in development.

**Supplementary Information:**

The online version contains supplementary material available at 10.1007/s10928-026-10037-8.

## Introduction

Therapeutic antibody dosing regimens are often limited by various molecular, formulation, and administration factors. Intravenous (IV) administration has long been the dominant route, with dosing intervals largely dictated by antibody half-life [1]. Although these regimens are typically optimized for efficacy, there is potential to improve other aspects of the target product profile (TPP) [2].

Subcutaneous (SC) administration has emerged as an attractive alternative, offering advantages such as improved convenience, reduced infusion-related reactions, shorter administration times, and potentially more favorable pharmacokinetic (PK) profiles (e.g., higher C_min_ or C_avg_ and lower C_max_) [3]. These benefits make SC delivery appealing for both patients and providers [4]. However, SC administration introduces its own limitations—most notably, injection volume [5]. Rapid SC injection is typically restricted to approximately two milliliters (mL) or less due to tissue resistance to bulk fluid flow, thus requiring lower doses and more frequent schedules compared to IV regimens. These constraints can lead to suboptimal TPPs when therapeutic windows allow for more flexible dosing [6].

**Fig. 1:**
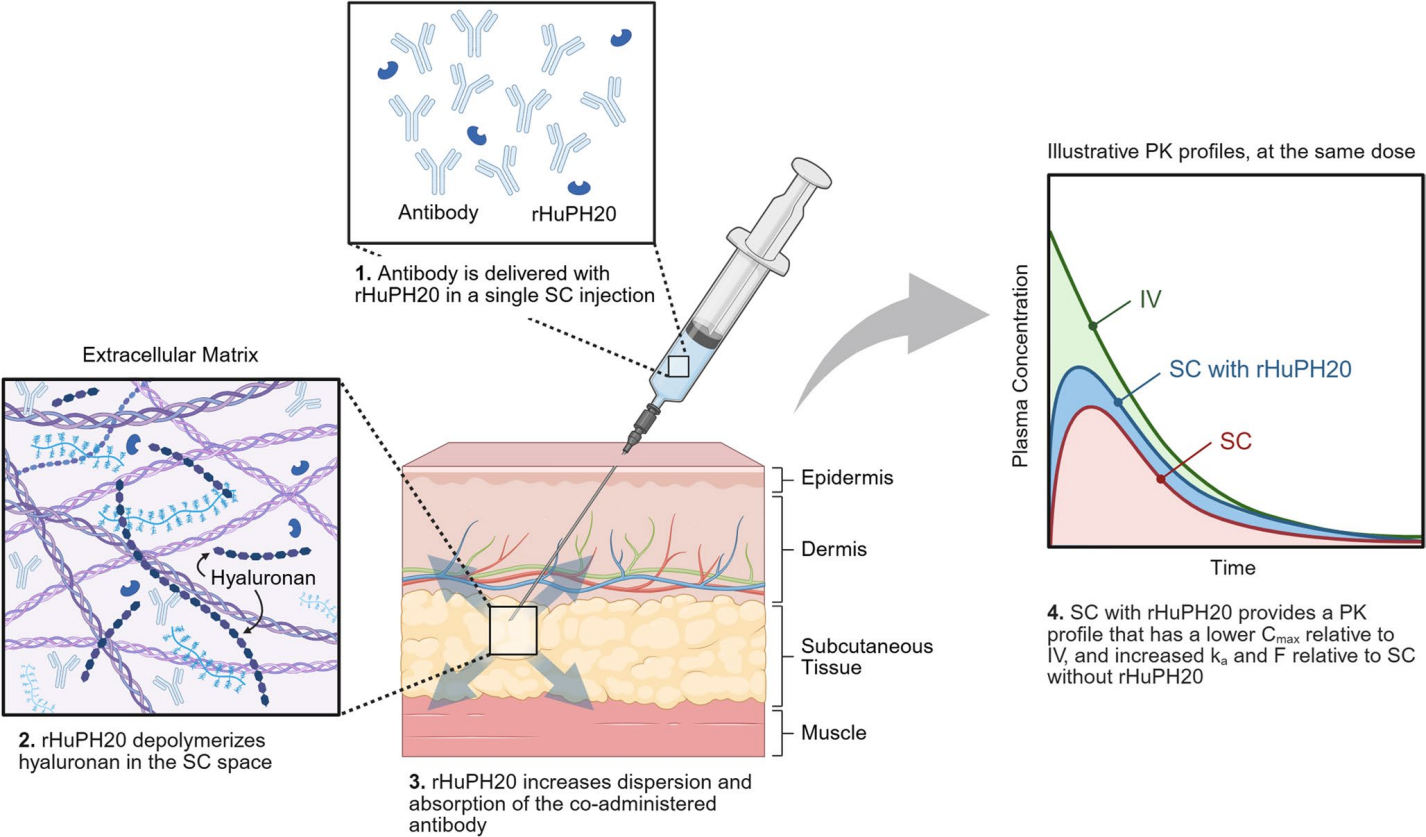
Schematic illustrating an antibody administered subcutaneously with rHuPH20. rHuPH20 enzymatically depolymerizes subcutaneous hyaluronan, transiently increasing tissue permeability and thereby enabling higher‑volume, faster SC injections. This mechanism is linked to its effects on absorption rate (k_a_) and bioavailability (F). The plot compares illustrative IV, SC, and SC with rHuPH20 concentration–time profiles, highlighting differences in C_max_, C_avg_, and C_min_ that inform feasibility and regimen‑selection considerations

Recent innovations have expanded the SC delivery landscape, including high-concentration formulations, half-life extension technologies, and enzymatic approaches such as recombinant human hyaluronidase PH20 (rHuPH20, Halozyme Therapeutics, Inc.) [3, 5]. rHuPH20 depolymerizes hyaluronan in the SC space, enabling rapid administration of high-dose, high-volume therapeutics [7] (see Fig. 1). rHuPH20 is co-administered with ten approved biologics across oncology and autoimmune indications [8–17]. Nine of these are co-formulated monoclonal antibody products, which span a broad range of SC dosing scenarios, with doses ranging from 600 to 1,875 mg, delivery volumes from 5 to 23 mL, and injection times from 20 s to 10 min. The remaining product, HyQvia—polyclonal IgG—represents the extreme end of the spectrum, administered SC at doses up to 60,000 mg in 600 mL at infusion rates of 5 mL/min. This breadth underscores rHuPH20’s ability to overcome historical constraints on SC injection volume and speed.

Despite these advances, quantifying the strategic impact of SC-enabling technologies is complex. Traditional PK simulations provide valuable insights for evaluation but are typically presented in formats that are limited in scope and difficult to fully leverage for dose regimen design decisions. This gap highlights the need for tools that translate complex PK modeling analyses into intuitive, decision-ready outputs. Such approaches should enable cross-functional teams—including clinicians and commercialization teams—to evaluate trade-offs between dose, frequency, and delivery feasibility early in development.

The objective of this work is to present a PK‑based visual framework—Operating Space Maps—that evaluates SC feasibility with rHuPH20 under exposure‑based criteria (C_avg_ or C_min_) and supports dose‑regimen design across a broad range of scenarios.

## Methods

### Dose regimen landscape

Data to characterize the dose regimen landscape of antibody therapeutics were obtained from two sources, approved product labels available through DailyMed [18] and PharmaCircle [19]. Dosage information was extracted from each product label using the GPT 4o mini large language model (LLM). To reduce the likelihood of extraction errors or hallucinations, each label was processed independently five times, and only dose regimens that appeared consistently in at least four of the five extraction outputs were retained for analysis.

From this database, only antibodies administered IV or SC with a worldwide status of marketed, approved, filed, phase 3, phase 2, and phase 1 as of January 1, 2025, were included. For each antibody, dosing regimens—defined by the combination of dose route, amount and frequency—were harmonized and recorded as distinct entries. For example, daratumumab is marketed both as an IV product (Darzalex^®^) and as a SC co-formulation with rHuPH20 (Darzalex Faspro™), each with multiple treatment-stage–dependent schedules, resulting in eight regimen entries in the database. Regimen strings were canonicalized and duplicate records at the drug–indication level were removed. When multiple source records mapped to the same regimen, the entry with the most advanced regulatory status was retained (approved > phase 3 > phase 2 > phase 1), and biosimilars were retained as distinct products. Entries corresponding to rHuPH20-enabled products were human-verified to ensure accurate regimen capture. All doses in mg/kg were converted to mg by multiplying by 70 kg. The resulting database consisted of 669 products and 1704 dosing unique regimen.

### Antibody PK simulations

**Fig. 2:**
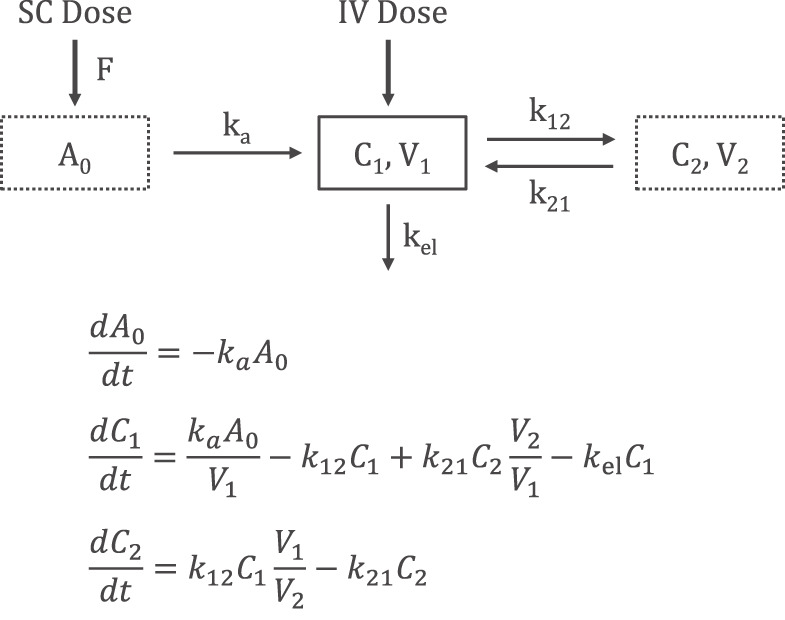
Two‑compartment model with first‑order SC absorption. The SC depot (A_0_) and central and peripheral compartments (C_1_, C_2_) are shown along with the rate constants (k_a_, k_el_, k_12_, k_21_), the compartment volumes (V_1_, V_2_), and the SC bioavailability (F). The accompanying differential equations describe the time evolution of each state variable

A two-compartment model with first-order SC absorption was used to simulate representative antibody pharmacokinetics. The model comprises a SC depot amount (A_0_) and central and peripheral compartment concentrations (C_1_ and C_2_, respectively). Parameters include central and peripheral volumes (V_1_, V_2_), absorption and elimination rate constants (k_a_, k_el_), inter-compartmental distribution rate constants (k_12_, k_21_), and SC bioavailability (F). The corresponding differential equations and parameter values are shown in Fig. 2; Table 1, respectively; these values were derived from a previously published PopPK analysis of ten clinical antibodies [20], with k_a_ and F adjusted for rHuPH20 simulations according to that analysis.

**Table 1:** Parameter definitions and values for the two‑compartment model with first‑order SC absorption, including corresponding rHuPH20‑adjusted parameters

Parameter	Description	Value	Units
V_1_	Volume of distribution for central compartment	2.85	L
V_2_	Volume of distribution for peripheral compartment	2.85	L
k_a_	Absorption rate from SC depot	0.28	1/day
k_el_	Elimination rate from central compartment	0.08	1/day
k_12_	Distribution rate from central → peripheral	0.22	1/day
k_21_	Distribution rate from peripheral → central	0.22	1/day
F	SC bioavailability	0.69	fraction
k_aPH20_	Absorption rate from SC depot when administered with rHuPH20	0.35	1/day
F_PH20_	SC bioavailability when administered with rHuPH20	0.76	fraction

SC doses (mg) were converted to nmol using 10^6^/MW (assuming an antibody molecular weight (MW) of 150,000 g/mol) multiplied by F, and added as a bolus to A_0_. IV doses (mg) were converted to nmol and then to nM by dividing by V_1_, and added as a bolus to C_1_. Dose administrations followed the labeled schedule times (e.g., QW, Q2W, Q4W, Q8W, Q12W, Q24W). For each regimen, steady-state PK metrics (e.g., C_avg, ss_) were defined over the final dosing interval for that regimen. Dose-matching calculations assumed PK linearity across all dose levels.

All simulations were performed using MATLAB^®^ (Mathworks, Natick, MA). Ordinary differential equations (ODEs) were solved in using ode15s with default tolerances, integrating segment‑by‑segment between dose times on a daily output grid (1‑day step). Implementation details and the full simulation configuration are provided in Supplementary Code S1.

These generic parameters serve as a representative example to illustrate the methodology and provide a starting point for assessing broad PK trends and guiding early strategic decisions. Asset-specific modeling can be performed subsequently to support more detailed assessments.

### Operating space maps

The term “Operating Space Map” used in this work refers to a visual framework composed of multiple antibody PK simulations, each representing a distinct dosing regimen. These simulations are systematically organized to allow the viewer to intuitively navigate between regimens and observe how specific changes in dose or frequency influence the resulting therapeutic profile and administration options.

To create an antibody dose regimen Operating Space Map, the workflow summarized in Table 2 was used. First, a benchmark dosing regimen was defined, such as 1000 mg IV. A benchmark regimen refers to a reference dose and route of administration that serves as the starting point for comparison of alternative dosing strategies. Second, simulations were generated for this benchmark regimen at schedules of every 1, 2, 4, 8, 12, and 24 weeks (QW, Q2W, Q4W, Q8W, Q12W, and Q24W). Within an Operating Space Map, these benchmark regimens are shown on the vertical axis and represented with red PK curves. Third, for each benchmark regimen, a set of simulations were generated for a new alternative regimen—SC administration with rHuPH20—across the same range of schedules, with the SC antibody dose defined to match the average steady-state concentration (C_avg_) of the corresponding benchmark regimen. Theses SC regimens with rHuPH20 are shown on the horizontal axis and represented with blue PK curves, with the corresponding dose displayed in the title of each plot. Finally, the background of each plot is color-coded to indicate the injection volume required for the SC dose with rHuPH20, assuming a 150 mg/mL antibody formulation. This visual cue enables rapid assessment of practical delivery options based on device constraints. The ranges were delineated based on the capacities of hand-held SC delivery devices, such as autoinjectors, for which rapid administration can be accommodated with rHuPH20. The PK profiles are represented in nM; however, conversion to µg/mL can be accomplished by multiplying by 0.15, assuming a typical molecular weight of 150,000 g/mol.

**Table 2:** Steps for building an antibody operating space map, from benchmark PK simulation to alternative‑regimen dose matching and visualization

Step	Description	Example
1	Define benchmark regimen	1000 mg IV
2	Simulate benchmark PK across schedules	QW, Q2W, Q4W, Q8W, Q12W, Q24W
3	Select matching PK metric	C_avg, ss_
4	Specify alternative regimen	SC + rHuPH20
5	Solve for alternative-regimen, metric-matched doses	1000 mg Q4W → 340 mg QW
6	Convert doses to volumes	340 mg ÷ 150 mg/mL = 2.3 mL
7	Apply volume-based color bands	0–2.25 mL; 2.25–10 mL; 10–20 mL; >20 mL
8	Overlay benchmark and alternative PK profiles	IV and SC + rHuPH20 PK curves overlaid
9	Interpret regimen trade-offs	↓ C_max, ss_; ↑ C_avg, ss_; ↓ frequency; etc.

The examples provided are based on matching the average steady-state concentration (C_avg_) of the benchmark regimen. However, the analysis could analogously be conducted by matching the minimum steady-state concentration (C_min_), or any other PK or PD metric that is known to drive a response (e.g., target occupancy).

## Results

### Landscape of IV and SC operating spaces

**Fig. 3:**
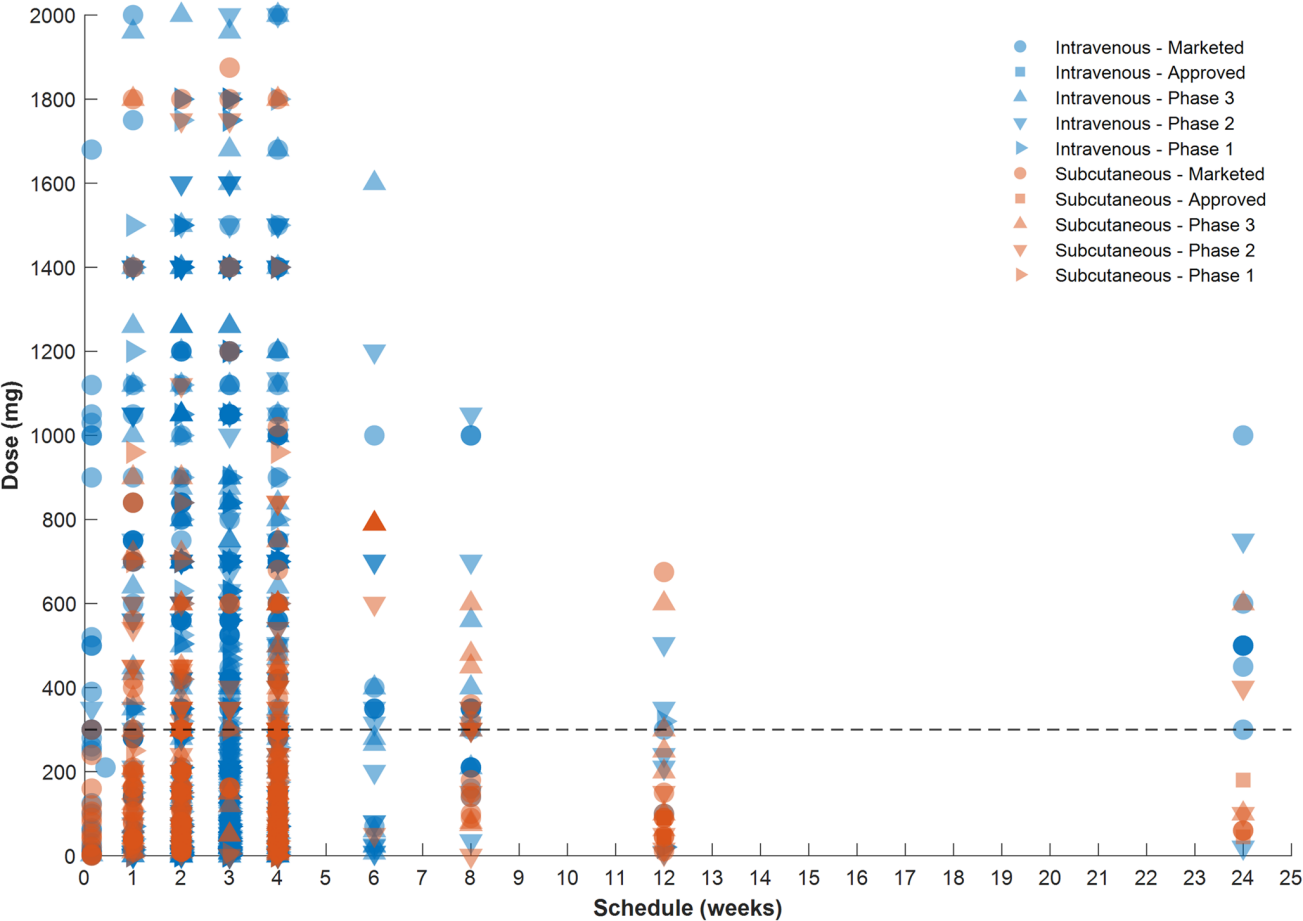
Landscape of IV and SC dosing regimens, from clinical development through approval. Data sourced from DailyMed and PharmaCircle, current as of January 1, 2025. Each symbol represents one of the 1704 unique dosing regimens across 669 antibodies. The horizontal dashed line indicates the approximate upper limit for SC delivery using a 2.25 mL autoinjector, corresponding to the maximum volume typically feasible for a 150 mg/mL formulation

Figure 3 illustrates the current dosing regimens for all IV and SC antibody therapeutics, spanning clinical development through marketed products. Most antibodies, irrespective of route of administration, are delivered at monthly schedules or less frequently; 94% of IV and 81% of SC regimens fall within this range. The differences in dose levels exemplify the volume constraints imposed by typical (i.e., non-rHuPH20 facilitated) SC delivery relative to IV; for IV regimens, 78% are less than or equal to 1000 mg and 49% less than or equal to 300 mg, while for SC regimens, 96% are less than or equal to 1000 mg and 75% less than or equal to 300 mg. For IV, the percentage of regimens that are less than or equal to 300 mg at phase 1, phase 2, phase 3, approved, and marketed are 59% (215/366), 52% (180/346), 42% (116/276), 44% (7/16), and 35% (89/254), respectively, while for SC these percentages are 59% (16/27), 70% (74/106), 63% (69/110), 94% (30/32), and 84% (143/171).

### Operating space maps

**Fig. 4:**
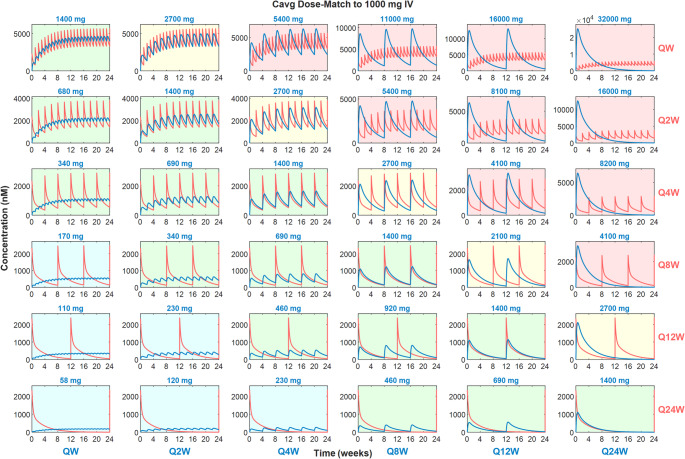
Operating Space Map for a generic antibody comparing a benchmark regimen of 1000 mg IV to SC regimens with rHuPH20. Each graph displays the PK profile for the SC regimen with rHuPH20 (blue curve) at the schedule indicated by the column, with the dose (shown in the blue title) calculated to match the average steady-state concentration (C_avg_) of the benchmark PK profile (red curve) at the schedule indicated by the row. Background plot colors:  0 – 2.25 mL,  2.25 – 10 mL,  10 – 20 mL,  > 20 mL

**Fig. 5:**
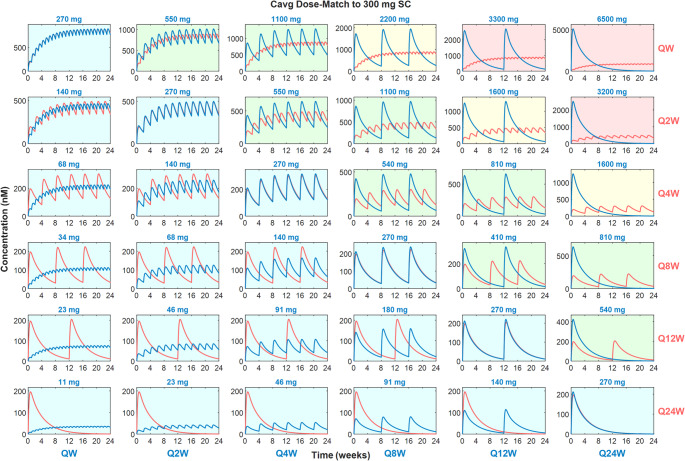
Operating Space Map for a generic antibody comparing a benchmark regimen of 300 mg SC (without rHuPH20) to SC regimens with rHuPH20. Each graph displays the PK profile for the SC regimen with rHuPH20 (blue curve) at the schedule indicated by the column, with the dose (shown in the blue title) calculated to match the average steady-state concentration (C_avg_) of the benchmark PK profile (red curve) at the schedule indicated by the row. Background plot colors:  0 – 2.25 mL,  2.25 – 10 mL,  10 – 20 mL,  > 20 mL

**Fig. 6:**
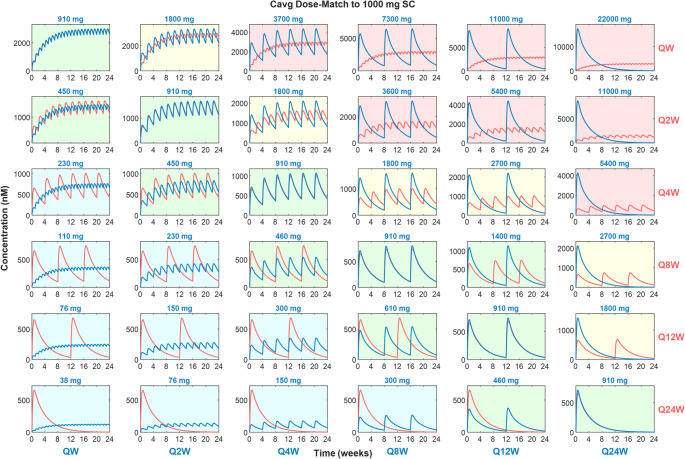
Operating Space Map for a generic antibody comparing a benchmark regimen of 1000 mg SC (without rHuPH20, assuming theoretically possible, e.g., delivered as a slow infusion) to SC regimens with rHuPH20. Each graph displays the PK profile for the SC regimen with rHuPH20 (blue curve) at the schedule indicated by the column, with the dose (shown in the blue title) calculated to match the average steady-state concentration (C_avg_) of the benchmark PK profile (red curve) at the schedule indicated by the row. Background plot colors:  0 – 2.25 mL,  2.25 – 10 mL,  10 – 20 mL,  > 20 mL

Applying the aforementioned PK model, simulations were generated for a few typical benchmark dosing regimens of 1000 mg IV, 300 mg SC (without rHuPH20), and 1000 mg SC (without rHuPH20, assuming theoretically possible, e.g., delivered as a slow infusion), and are depicted as Operating Space Maps in Figs. 4 and 5, and 6, respectively. These maps present the PK profiles for each benchmark regimen along with the corresponding C_avg_-matched PK profiles for SC regimens with rHuPH20. All maps have a consistent layout and provide a means to evaluate the feasibility of implementing various regimens based on dose and volume constraints. Specifically, areas of the maps that contain plots with a blue background represent SC antibody doses (with rHuPH20) that can be delivered in a volume less than 2.25 mL (i.e., the limits of a standard autoinjector), assuming a typical formulation of 150 mg/mL. These scenarios do not necessarily require rHuPH20 for SC delivery and represent the typical, constrained SC operating space. Areas of the maps that contain plots with green, yellow, and red backgrounds represent the expanded SC operating spaces afforded by the ability of rHuPH20 to overcome delivery constraints and allow rapid injection of volumes greater than 2.25 mL.

Because rHuPH20 is a biologic enzyme, it is generally compatible with most antibody formulations and typically requires no major formulation changes. While not strictly necessary, increasing the antibody concentration during the transition from IV to SC delivery (e.g., from 20 mg/mL to 150 mg/mL) is often preferred to minimize injection volume and improve administration feasibility.

Specific applications of the Operating Space Maps are discussed in the following examples.

### Evaluating IV to SC conversion

Figure 4 depicts an Operating Space Map of options for converting from a benchmark regimen of 1000 mg IV to SC with rHuPH20. Each row captures the 1000 mg IV dose at the corresponding schedule. For illustrative purposes, consider the third row for a common IV schedule of Q4W. The red curves in this row are all the same and exhibit maximum (C_max_), average (C_avg_), and minimum (C_min_) antibody concentrations of roughly 3000, 1000, and 500 nM, respectively. To achieve a SC regimen with an equivalent C_avg_ as the IV benchmark, i.e., approximately 1000 nM, and the same schedule of Q4W, the corresponding SC antibody dose (with rHuPH20) is 1400 mg. Assuming a typical formulation of 150 mg/mL, this corresponds to a delivery volume of 9 mL, demonstrating the need for rHuPH20 to achieve this dosing regimen as a rapid injection. One important feature of depicting the Operating Space Maps with PK curves is exemplified in this plot. The C_max_ for the SC with rHuPH20 regimen is roughly half that of the equivalent IV regimen, despite having the same C_avg_. For situations where C_max_ is the driver of toxicity and C_avg_ is the driver of efficacy, such scenarios become advantageous opportunities to improve the TPP through a safer and/or more efficacious regimen, in addition to the other benefits that SC administration affords over IV administration.

Continuing with this example, consider moving one graph to the right in Fig. 4 to a SC with rHuPH20 dosing regimen of Q8W. In this scenario, not only does the SC PK curve achieve the same C_avg_ and lower C_max_ than the IV benchmark, but the dosing interval is extended 2-fold. Enabling the reduction in dosing frequency while maintaining the safety and efficacy profiles not only improves a TPP but also creates potential competitive differentiation.

For each benchmark–alternative regimen pair shown in the Operating Space Maps, noncompartmental metrics—AUCτ, C_avg, ss_, C_min, ss_, C_max, ss_, and fluctuation index ((C_max, ss_–C_min, ss_)/C_avg, ss_)— were computed over the final dosing interval. Complete tables for all schedules are provided in the Supplementary Tables S1–S3.

### Evaluating SC dose interval extension

Figure 5 depicts the scenario of starting with a 300 mg SC (without rHuPH20) benchmark. This value is chosen because at a 150 mg/mL formulation the resulting volume of 2 mL can be easily delivered in a standard 2.25 mL autoinjector. For subcutaneous doses exceeding this threshold, injection volumes greater than 2 mL substantially increase tissue backpressure, generally necessitating co-administration with rHuPH20 to facilitate a more practical and rapid delivery. This concept is visually apparent in the Operating Space Map, where all plots above and to the right of the diagonal have background colors other than blue, emphasizing that if an extension of the existing (benchmark) SC dosing schedule is desired, then those regimens delivered as a rapid injection are viable by co-administration with rHuPH20.

If a similar scenario as described previously is considered, i.e., extending a current SC (without rHuPH20) regimen of 300 mg Q4W to SC with rHuPH20 at Q8W, an antibody dose of 540 mg is required. Unlike the IV benchmark example, the new (blue) PK curves demonstrate a higher C_max_ and lower C_min_ than the benchmark SC condition, while maintaining an equivalent C_avg_. This distinction is important, as the ability to accommodate these larger swings between C_max_ and C_min_ will be dependent on the therapeutic window of the antibody. In most cases, a move one or two graphs to the right of the benchmark schedule is likely within tolerable limits (i.e., concentrations evaluated in initial dose escalation studies), but as one moves further to the right on the map, these differences become more significant.

Figure 6 depicts the Operating Space Map if the benchmark SC dose (without rHuPH20) is increased to 1000 mg. This map is similar to the previous one in Fig. 3, but with a downward shift in the plot background colors, further emphasizing the expanded dose regimen options that are restricted without rHuPH20 and enabled with it, particularly when higher antibody concentrations (e.g., C_avg_) are required for disease modulation.

### External validation of simulations with rHuPH20

Corroboration of the qualitative trends shown in the Operating Space maps was performed using the PK of a recently approved rHuPH20‑coformulated product. SC ocrelizumab (Ocrevus Zunovo™), is dosed at 920 mg in 23 mL over approximately 10 min and exhibits similar overall exposure compared to IV ocrelizumab with a lower C_max_ (132 µg/mL for SC vs. ~ 200 µg/mL for IV) [14, 21]. These label‑supported trends—comparable C_avg_ (AUC), reduced C_max_, and feasible rapid SC delivery at high-dose/high-volume—align with the qualitative PK and administration patterns produced by the simulations.

## Discussion

SC antibody regimens are often constrained by formulation and volume, requiring lower doses and more frequent administration than is optimal for patients or health systems. These constraints can lead to suboptimal TPPs when therapeutic windows may allow for more flexible dosing. Model-informed evaluation can quantify the potential value of SC-enabling technologies; however, simulation outputs are often difficult for non-modelers to interpret. This work addresses this gap by introducing Operating Space Maps—a visual framework that organizes various PK simulations into an intuitive, navigable landscape of dose–frequency options that can guide strategic decision-making early in development.

The modeling framework in this work covered two scenarios. First, when converting an IV regimen to SC with rHuPH20, the maps show how equivalent exposure (e.g., C_avg_ or C_min_) can be maintained at the same or a less frequent schedule while reducing peak concentrations, which is advantageous in the case of C_max_-based toxicities. Second, for drugs already dosed SC without rHuPH20, the maps clarify how rHuPH20 can extend dose intervals by circumventing the standard rapid injection volume limit of ~ 2 mL, while preserving overall exposure.

Related visualization approaches, such as heatmaps or isometric exposure–response surfaces, are useful for summarizing how exposure metrics vary across dose and interval combinations. However, these tools generally do not incorporate the underlying PK‑profile time courses or the device and volume constraints that determine whether a regimen is practically deliverable by the SC route. By overlaying simulated PK profiles with SC administration limits (e.g., injection volume, concentration, delivery time), the Operating Space Maps provide an integrated view that enables identification of regimens that are both pharmacokinetically suitable and feasible to administer.

Although this case study focused on rHuPH20, the methodology is applicable to a broad set of SC-enabling technologies (e.g., high concentration formulations or half-life extension). The same mapping approach can be applied to match alternative PK/PD drivers (e.g., target occupancy) and to visualize how technology choices expand or constrain feasible dosing spaces.

Finally, several practical considerations shape how to use the framework. The simulations in this work employ generic, representative parameters derived from prior analyses to illustrate methodology and broad trends; they provide a starting point for strategic discussions but could be complemented by asset-specific modeling when making program decisions. Incorporating mechanistic details determined during drug development (e.g. target-mediated drug disposition or physiologically-based PK modeling frameworks) and integrating emerging clinical data to calibrate and validate the operating space predictions can further improve accuracy and strategic value. Additionally, this work used representative mean parameters without inter-individual variability (IIV); consequently, the figures display deterministic profiles rather than prediction intervals. As population variability and covariance structures become available for a given asset, the same workflow can incorporate IIV (e.g., Monte Carlo propagation of k_a_, k_el_, V_1_, V_2_, F) to produce prediction intervals and quantify uncertainty around C_max, ss_, C_avg, ss_, and C_min, ss_. In summary, Operating Space Maps transform model outputs into clear, decision-ready visuals that connect PK behavior to delivery feasibility and clinical strategy. They enable development teams—especially those including non-modelers—to navigate the dose–frequency landscape, appraise tradeoffs, and select regimens that align with efficacy, safety, convenience, and competitive goals. In practice, the maps also serve as a front‑end triage tool: teams can identify dose–interval regions that meet PK targets and satisfy SC feasibility constraints, and then advance only those short‑listed regimens to asset‑specific PopPK or TMDD modeling.

## Supplementary information

Below is the link to the electronic supplementary material.

Supplementary Material 1 (PDF 407 KB)

## Data Availability

The dose regimen landscape dataset was derived from DailyMed (public) and PharmaCircle (licensed) and is not available from the authors, but can be accessed from these sources subject to their terms.
