# Supplementary material for: Optimizing Subcutaneous Antibody Dosing Regimens Through Operating Space Maps: rHuPH20 Case Study
Source: J Pharmacokinet Pharmacodyn. 2026 Jun 29;53(4):33. doi: 10.1007/s10928-026-10037-8 (PMC13314913; doi:10.1007/s10928-026-10037-8)
Supplement: Supplementary file 1 — Supplementary Material 1 (PDF 407 KB) [file 10928_2026_10037_MOESM1_ESM.pdf]

Supplementary Material

Optimizing Subcutaneous Antibody Dosing Regimens Through Operating Space Maps: rHuPH20 Case Study

Journal of Pharmacokinetics and Pharmacodynamics

Ryan P. Nolan[1], Harish Chintakuntla[1]
[1] Halozyme Therapeutics, San Diego, CA, USA
publications@halozyme.com

Supplementary Tables: Tabulated data from example Operating Space Maps

For each benchmark regimen and its corresponding C_{avg}-matched SC+rHuPH20 regimen, steady-state pharmacokinetic metrics were calculated over the final dosing interval of the simulation. These included the average steady-state concentration ($C_{avg,ss}$), minimum steady-state concentration ($C_{min,ss}$), and maximum steady-state concentration ($C_{max,ss}$), each computed directly from the concentration–time values within the last dosing interval. The AUC over the dosing interval (AUC_{τ}) was obtained by trapezoidal numerical integration of the concentration profile across the same window, and Cavg,ss can equivalently be expressed as AUC_{τ} divided by the dosing interval length. The fluctuation index (FI) was calculated as $(C_{max,ss}–C_{min,ss})/C_{avg,ss}$, providing a normalized measure of peak–trough variability. All metrics were derived using the linear two-compartment PK model and steady-state windowing logic implemented in the MATLAB simulation code.

Supplementary Table S1 Tabulated data for the Operating Space Map comparing a benchmark regimen of 1000 mg IV to SC regimens with rHuPH20

1000 mg IV								SC with rHuPH20							
**Dose (mg)**	**Route**	**Schedule**	**$C_{avg,ss}$ (nM)**	**$C_{min,ss}$ (nM)**	**$C_{max,ss}$ (nM)**	**AUC_t (nM×day)**	**Fluctuation Index**	**Dose (mg)**	**Route**	**Schedule**	**$C_{avg,ss}$ (nM)**	**$C_{min,ss}$ (nM)**	**$C_{max,ss}$ (nM)**	**AUC_t (nM×day)**	**Fluctuation Index**
1000	IV	QW	3391	3321	4966	23736	0.49	1300	SC	QW	3513	3720	4298	24593	0.16
1000	IV	QW	3391	3321	4966	23736	0.49	2500	SC	Q2W	3665	3053	4625	51305	0.43
1000	IV	QW	3391	3321	4966	23736	0.49	5100	SC	Q4W	3876	2311	5990	108517	0.95
1000	IV	QW	3391	3321	4966	23736	0.49	10000	SC	Q8W	3857	1195	8862	216019	1.99
1000	IV	QW	3391	3321	4966	23736	0.49	15000	SC	Q12W	3872	587	12259	325277	3.01
1000	IV	QW	3391	3321	4966	23736	0.49	30000	SC	Q24W	3879	52	23545	651625	6.06
1000	IV	Q2W	1828	1429	3156	25592	0.94	640	SC	QW	1730	1832	2116	12107	0.16
1000	IV	Q2W	1828	1429	3156	25592	0.94	1300	SC	Q2W	1906	1588	2405	26678	0.43
1000	IV	Q2W	1828	1429	3156	25592	0.94	2500	SC	Q4W	1900	1133	2936	53195	0.95
1000	IV	Q2W	1828	1429	3156	25592	0.94	5100	SC	Q8W	1967	610	4519	110169	1.99
1000	IV	Q2W	1828	1429	3156	25592	0.94	7600	SC	Q12W	1962	297	6211	164807	3.01
1000	IV	Q2W	1828	1429	3156	25592	0.94	15000	SC	Q24W	1939	26	11773	325812	6.06
1000	IV	Q4W	945	534	2293	26461	1.86	320	SC	QW	865	916	1058	6054	0.16
1000	IV	Q4W	945	534	2293	26461	1.86	640	SC	Q2W	938	782	1184	13134	0.43
1000	IV	Q4W	945	534	2293	26461	1.86	1300	SC	Q4W	988	589	1527	27661	0.95
1000	IV	Q4W	945	534	2293	26461	1.86	2500	SC	Q8W	964	299	2215	54005	1.99
1000	IV	Q4W	945	534	2293	26461	1.86	3800	SC	Q12W	981	149	3106	82404	3.01
1000	IV	Q4W	945	534	2293	26461	1.86	7600	SC	Q24W	983	13	5965	165078	6.06
1000	IV	Q8W	480	141	1914	26858	3.70	160	SC	QW	432	458	529	3027	0.16
1000	IV	Q8W	480	141	1914	26858	3.70	320	SC	Q2W	469	391	592	6567	0.43
1000	IV	Q8W	480	141	1914	26858	3.70	640	SC	Q4W	486	290	752	13618	0.95
1000	IV	Q8W	480	141	1914	26858	3.70	1300	SC	Q8W	501	155	1152	28082	1.99
1000	IV	Q8W	480	141	1914	26858	3.70	1900	SC	Q12W	490	74	1553	41202	3.01
1000	IV	Q8W	480	141	1914	26858	3.70	3800	SC	Q24W	491	7	2982	82539	6.06
1000	IV	Q12W	321	46	1823	26934	5.54	110	SC	QW	297	315	364	2081	0.16
1000	IV	Q12W	321	46	1823	26934	5.54	210	SC	Q2W	308	256	388	4310	0.43
1000	IV	Q12W	321	46	1823	26934	5.54	420	SC	Q4W	319	190	493	8937	0.95
1000	IV	Q12W	321	46	1823	26934	5.54	850	SC	Q8W	328	102	753	18362	1.99
1000	IV	Q12W	321	46	1823	26934	5.54	1300	SC	Q12W	336	51	1062	28191	3.01
1000	IV	Q12W	321	46	1823	26934	5.54	2500	SC	Q24W	323	4	1962	54302	6.06
1000	IV	Q24W	161	2	1780	27002	11.06	53	SC	QW	143	152	175	1003	0.16
1000	IV	Q24W	161	2	1780	27002	11.06	110	SC	Q2W	161	134	203	2257	0.43
1000	IV	Q24W	161	2	1780	27002	11.06	210	SC	Q4W	160	95	247	4468	0.95
1000	IV	Q24W	161	2	1780	27002	11.06	420	SC	Q8W	162	50	372	9073	1.99
1000	IV	Q24W	161	2	1780	27002	11.06	640	SC	Q12W	165	25	523	13878	3.01
1000	IV	Q24W	161	2	1780	27002	11.06	1300	SC	Q24W	168	2	1020	28237	6.06

Supplementary Table S2 Tabulated data for the Operating Space Map comparing a benchmark regimen of 300 mg SC (without rHuPH20) to SC regimens with rHuPH20.

300 mg SC								SC with rHuPH20							
Dose (mg)	Route	Schedule	$C_{avg,ss}$ (nM)	$C_{min,ss}$ (nM)	$C_{max,ss}$ (nM)	AUC_t (nM×day)	Fluctuation Index	Dose (mg)	Route	Schedule	$C_{avg,ss}$ (nM)	$C_{min,ss}$ (nM)	$C_{max,ss}$ (nM)	AUC_t (nM×day)	Fluctuation Index
300	SC	QW	737	793	892	5160	0.13	270	SC	QW	730	773	893	5108	0.16
300	SC	QW	737	793	892	5160	0.13	550	SC	Q2W	806	672	1017	11287	0.43
300	SC	QW	737	793	892	5160	0.13	1100	SC	Q4W	836	498	1292	23406	0.95
300	SC	QW	737	793	892	5160	0.13	2200	SC	Q8W	849	263	1950	47524	1.99
300	SC	QW	737	793	892	5160	0.13	3300	SC	Q12W	852	129	2697	71561	3.01
300	SC	QW	737	793	892	5160	0.13	6500	SC	Q24W	840	11	5101	141185	6.06
300	SC	Q2W	400	343	490	5600	0.37	140	SC	QW	378	401	463	2648	0.16
300	SC	Q2W	400	343	490	5600	0.37	270	SC	Q2W	396	330	499	5541	0.43
300	SC	Q2W	400	343	490	5600	0.37	550	SC	Q4W	418	249	646	11703	0.95
300	SC	Q2W	400	343	490	5600	0.37	1100	SC	Q8W	424	131	975	23762	1.99
300	SC	Q2W	400	343	490	5600	0.37	1600	SC	Q12W	413	63	1308	34696	3.01
300	SC	Q2W	400	343	490	5600	0.37	3300	SC	Q24W	427	6	2590	71679	6.06
300	SC	Q4W	208	127	304	5812	0.85	68	SC	QW	184	195	225	1286	0.16
300	SC	Q4W	208	127	304	5812	0.85	140	SC	Q2W	205	171	259	2873	0.43
300	SC	Q4W	208	127	304	5812	0.85	270	SC	Q4W	205	122	317	5745	0.95
300	SC	Q4W	208	127	304	5812	0.85	550	SC	Q8W	212	66	487	11881	1.99
300	SC	Q4W	208	127	304	5812	0.85	820	SC	Q12W	212	32	670	17782	3.01
300	SC	Q4W	208	127	304	5812	0.85	1600	SC	Q24W	207	3	1256	34753	6.06
300	SC	Q8W	105	34	224	5904	1.81	34	SC	QW	92	97	112	643	0.16
300	SC	Q8W	105	34	224	5904	1.81	68	SC	Q2W	100	83	126	1395	0.43
300	SC	Q8W	105	34	224	5904	1.81	140	SC	Q4W	106	63	164	2979	0.95
300	SC	Q8W	105	34	224	5904	1.81	270	SC	Q8W	104	32	239	5833	1.99
300	SC	Q8W	105	34	224	5904	1.81	410	SC	Q12W	106	16	335	8891	3.01
300	SC	Q8W	105	34	224	5904	1.81	820	SC	Q24W	106	1	644	17811	6.06
300	SC	Q12W	71	11	205	5927	2.75	23	SC	QW	62	66	76	435	0.16
300	SC	Q12W	71	11	205	5927	2.75	45	SC	Q2W	66	55	83	923	0.43
300	SC	Q12W	71	11	205	5927	2.75	91	SC	Q4W	69	41	107	1936	0.95
300	SC	Q12W	71	11	205	5927	2.75	180	SC	Q8W	69	22	160	3888	1.99
300	SC	Q12W	71	11	205	5927	2.75	270	SC	Q12W	70	11	221	5855	3.01
300	SC	Q12W	71	11	205	5927	2.75	550	SC	Q24W	71	1	432	11946	6.06
300	SC	Q24W	35	0	196	5936	5.54	11	SC	QW	30	31	36	208	0.16
300	SC	Q24W	35	0	196	5936	5.54	23	SC	Q2W	34	28	43	472	0.43
300	SC	Q24W	35	0	196	5936	5.54	45	SC	Q4W	34	20	53	958	0.95
300	SC	Q24W	35	0	196	5936	5.54	91	SC	Q8W	35	11	81	1966	1.99
300	SC	Q24W	35	0	196	5936	5.54	140	SC	Q12W	36	5	114	3036	3.01
300	SC	Q24W	35	0	196	5936	5.54	270	SC	Q24W	35	0	212	5865	6.06

Supplementary Table S3 Tabulated data for the Operating Space Map comparing a benchmark regimen of 1000 mg SC (without rHuPH20) to SC regimens with rHuPH20.

1000 mg SC								SC with rHuPH20							
**Dose (mg)**	**Route**	**Schedule**	**$C_{avg,ss}$ (nM)**	**$C_{min,ss}$ (nM)**	**$C_{max,ss}$ (nM)**	**AUC_t (nM×day)**	**Fluctuation Index**	**Dose (mg)**	**Route**	**Schedule**	**$C_{avg,ss}$ (nM)**	**$C_{min,ss}$ (nM)**	**$C_{max,ss}$ (nM)**	**AUC_t (nM×day)**	**Fluctuation Index**
1000	SC	QW	2457	2644	2974	17199	0.13	910	SC	QW	2459	2604	3009	17215	0.16
1000	SC	QW	2457	2644	2974	17199	0.13	1800	SC	Q2W	2639	2198	3330	36939	0.43
1000	SC	QW	2457	2644	2974	17199	0.13	3600	SC	Q4W	2736	1631	4228	76600	0.95
1000	SC	QW	2457	2644	2974	17199	0.13	7300	SC	Q8W	2816	873	6469	157694	1.99
1000	SC	QW	2457	2644	2974	17199	0.13	11000	SC	Q12W	2840	431	8990	238537	3.01
1000	SC	QW	2457	2644	2974	17199	0.13	22000	SC	Q24W	2844	38	17267	477858	6.06
1000	SC	Q2W	1333	1143	1632	18668	0.37	450	SC	QW	1216	1288	1488	8513	0.16
1000	SC	Q2W	1333	1143	1632	18668	0.37	910	SC	Q2W	1334	1111	1683	18675	0.43
1000	SC	Q2W	1333	1143	1632	18668	0.37	1800	SC	Q4W	1368	816	2114	38300	0.95
1000	SC	Q2W	1333	1143	1632	18668	0.37	3600	SC	Q8W	1389	430	3190	77767	1.99
1000	SC	Q2W	1333	1143	1632	18668	0.37	5500	SC	Q12W	1420	215	4495	119268	3.01
1000	SC	Q2W	1333	1143	1632	18668	0.37	11000	SC	Q24W	1422	19	8633	238929	6.06
1000	SC	Q4W	692	424	1015	19375	0.85	230	SC	QW	622	658	760	4351	0.16
1000	SC	Q4W	692	424	1015	19375	0.85	450	SC	Q2W	660	550	832	9235	0.43
1000	SC	Q4W	692	424	1015	19375	0.85	910	SC	Q4W	692	412	1069	19363	0.95
1000	SC	Q4W	692	424	1015	19375	0.85	1800	SC	Q8W	694	215	1595	38883	1.99
1000	SC	Q4W	692	424	1015	19375	0.85	2700	SC	Q12W	697	106	2207	58550	3.01
1000	SC	Q4W	692	424	1015	19375	0.85	5500	SC	Q24W	711	9	4317	119465	6.06
1000	SC	Q8W	351	112	746	19679	1.81	110	SC	QW	297	315	364	2081	0.16
1000	SC	Q8W	351	112	746	19679	1.81	230	SC	Q2W	337	281	425	4720	0.43
1000	SC	Q8W	351	112	746	19679	1.81	450	SC	Q4W	342	204	529	9575	0.95
1000	SC	Q8W	351	112	746	19679	1.81	910	SC	Q8W	351	109	806	19658	1.99
1000	SC	Q8W	351	112	746	19679	1.81	1400	SC	Q12W	361	55	1144	30359	3.01
1000	SC	Q8W	351	112	746	19679	1.81	2700	SC	Q24W	349	5	2119	58646	6.06
1000	SC	Q12W	235	37	684	19758	2.75	76	SC	QW	205	218	251	1438	0.16
1000	SC	Q12W	235	37	684	19758	2.75	150	SC	Q2W	220	183	277	3078	0.43
1000	SC	Q12W	235	37	684	19758	2.75	300	SC	Q4W	228	136	352	6383	0.95
1000	SC	Q12W	235	37	684	19758	2.75	610	SC	Q8W	235	73	541	13177	1.99
1000	SC	Q12W	235	37	684	19758	2.75	910	SC	Q12W	235	36	744	19733	3.01
1000	SC	Q12W	235	37	684	19758	2.75	1800	SC	Q24W	233	3	1413	39097	6.06
1000	SC	Q24W	118	2	655	19791	5.55	38	SC	QW	103	109	126	719	0.16
1000	SC	Q24W	118	2	655	19791	5.55	76	SC	Q2W	111	93	141	1560	0.43
1000	SC	Q24W	118	2	655	19791	5.55	150	SC	Q4W	114	68	176	3192	0.95
1000	SC	Q24W	118	2	655	19791	5.55	300	SC	Q8W	116	36	266	6481	1.99
1000	SC	Q24W	118	2	655	19791	5.55	450	SC	Q12W	116	18	368	9758	3.01
1000	SC	Q24W	118	2	655	19791	5.55	910	SC	Q24W	118	2	714	19766	6.06

Supplementary Code S1 MATLAB scripts used to implement the two-compartment pharmacokinetic model, perform steady-state dose matching between benchmark and SC+rHuPH20 regimens across dosing schedules, and generate the corresponding operating space map visualizations with volume annotations.

```
%% ========================================================================
%  Two-Compartment PK Simulation and Dose-Matching Visualization
%  (e.g. Baseline SC vs. SC + rHuPH20-like absorption enhancement)
%  ------------------------------------------------------------------------
%  Purpose
%    • Simulate a linear two-compartment PK model with first-order SC input.
%    • For each pair of dosing schedules, compute the SC dose under an
%      rHuPH20-like condition that matches the steady-state average plasma
%      concentration (Cavg,ss) of a baseline regimen (here SC without rHuPH20).
%    • Visualize baseline vs. dose-matched profiles in a 6×6 grid and tint
%      backgrounds by estimated autoinjector (AI) volume bins.
%
%  Model (units)
%    Time: days
%    Dose: mg (converted internally to nmol via *1e6 / MW)
%    SC depot amount: nmol
%    Plasma/peripheral concentrations: nM
%    y(1) = A_sc   (nmol)  : SC depot
%    y(2) = Cc     (nM)    : central (plasma)
%    y(3) = Cp     (nM)    : peripheral
%    ODEs:
%      dA_sc/dt = -ka * A_sc
%      dCc/dt   =  ka*A_sc/Vc - k12*Cc + k21*Cp*(Vp/Vc) - kel*Cc
%      dCp/dt   =  k12*Cc*(Vc/Vp) - k21*Cp
%
%  Assumptions
%    • Linear PK with constant parameters.
%    • SC bioavailability (F) and absorption rate (ka) are route-specific.
%    • rHuPH20-like setting increases ka and F modestly (illustrative).
%    • Cavg,ss window = final dosing-interval days for each schedule.
%    • AI volume estimate assumes 150 mg/mL: volume (mL) = dose(mg)/150.
%
%  Figure
%    • Rows: baseline SC schedules
%    • Columns: SC + rHuPH20 schedules (dose-matched to row baseline Cavg,ss)
%    • Red:   baseline SC at 1000 mg
%    • Blue:  SC + rHuPH20 scaled to matched dose
%    • Background tint: AI volume bin
%
%  MATLAB
%    • Requires R2016b+ (local functions in scripts).
%
%  ========================================================================


%% Housekeeping
```

```
clear; clc; close all;


%% -------------------------- Parameters -----------------------------------
% Core PK parameters
p.Vc   = 2.85;     % L (central volume)
p.Vp   = 2.85;     % L (peripheral volume)
p.CL   = 0.23;     % L/day (clearance)
p.Q    = 0.62;     % L/day (inter-compartmental CL)
p.ka   = 0.28;     % 1/day (SC absorption rate)
p.F    = 0.69;     % fraction (SC bioavailability)
p.MW   = 150000;   % g/mol (approx. mAb)


% Derived rate constants (1/day)
p.kel  = p.CL / p.Vc;
p.k12  = p.Q  / p.Vc;
p.k21  = p.Q  / p.Vp;


%% ------------------------- Simulation Setup ------------------------------
% Time grid (days)
duration_days = 48*7;                 % 48 weeks
t_meas_days   = (0:1:duration_days).';% daily grid


% Baseline regimen
baseline_dose_mg  = 1000;   % mg
baseline_route    = 'SC';   % baseline route
formulation_mg_mL = 150;    % for AI volume estimate only


% Schedules of interest (rows and columns)
schedules    = {'QW','Q2W','Q4W','Q8W','Q12W','Q24W'};
interval_day = [   7,   14,    28,    56,     84,    168];


% rHuPH20-like adjustment (illustrative)
p_ph20      = p;
p_ph20.ka   = p.ka * (5/4);
p_ph20.F    = p.F * (1.1);


% Colors
c_blue = [0, 128, 199] ./ 256;
c_red  = [255, 100, 100] ./ 256;


% AI volume bins (mL) and panel colors (light tints)
ai_bins_mL     = [2.25, 10, 20];                         % (0-2.25], (2.25-10], (10-20]
```

```
ai_bin_colors  = [229 255 255; 229 255 229; 255 255 229] ./ 255;
overflow_color = [255 229 229] ./ 255;                   % >20 mL


% Figure layout
fig = figure('Color','w','Position',[420,120,1220,820]);
tl  = tiledlayout(6,6,'TileSpacing','compact','Padding','compact');
fs  = 10;


%% ----------------------- Main Simulation Loops ---------------------------
for i = 1:numel(schedules)     % Row: baseline SC schedule
    base_sched = schedules{i};


    % Baseline SC simulation (fixed 1000 mg)
    cfg_base            = struct();
    cfg_base.route      = baseline_route;
    cfg_base.schedule   = base_sched;
    cfg_base.dose_mg    = baseline_dose_mg;
    cfg_base.t_meas_days= t_meas_days;


    [t_base, y_base] = runPkModel(p, cfg_base);       % y_base(:,2) = Cc (nM)
    last_idx_base    = (numel(t_base) - interval_day(i) + 1) : numel(t_base);
    Cavg_base_ss     = mean(y_base(last_idx_base, 2), 'omitnan');


    for j = 1:numel(schedules) % Column: SC + rHuPH20 schedule to be dose-matched
        ph20_sched = schedules{j};


        % SC + rHuPH20 simulation (unscaled)
        cfg_ph20              = struct();
        cfg_ph20.route        = 'SC';
        cfg_ph20.schedule     = ph20_sched;
        cfg_ph20.dose_mg      = baseline_dose_mg;   % temporary; will scale
        cfg_ph20.t_meas_days  = t_meas_days;


        [t_ph20, y_ph20]  = runPkModel(p_ph20, cfg_ph20);
        last_idx_ph20     = (numel(t_ph20) - interval_day(j) + 1) : numel(t_ph20);
        Cavg_ph20_ss      = mean(y_ph20(last_idx_ph20, 2), 'omitnan');


        % Dose to match baseline Cavg,ss (linearity assumption)
        dose_match_mg = round(cfg_ph20.dose_mg * (Cavg_base_ss / Cavg_ph20_ss), 2,
'significant');
```

```
        % Plot tile
        nexttile(tl, (i-1)*numel(schedules) + j); hold on;


        % Baseline SC (fixed 1000 mg)
        plot(t_base./7, y_base(:,2), 'Color', c_red, 'LineWidth', 1.0);


        % SC + rHuPH20 scaled to matched dose
        scale_factor = dose_match_mg / cfg_ph20.dose_mg;
        plot(t_ph20./7, y_ph20(:,2) .* scale_factor, 'Color', c_blue, 'LineWidth', 1.0);


        % Title shows matched dose (mg) for column regimen
        title(sprintf('%.3g mg', dose_match_mg), 'Color', c_blue, 'FontSize', fs-1);


        % Axes: show first 24 weeks
        xlim([0, duration_days/2/7]);           % 0-24 weeks
        xticks(0:4:24);
        y_max = max([y_base(:,2); y_ph20(:,2).*scale_factor]);
        ylim([0, y_max*1.1]);


        % Column headers (top row): schedule labels for rHuPH20 condition
        if i == 1
            xl_top = xlim; yl_top = ylim;
            text(mean(xl_top), yl_top(2)*1.02, ph20_sched, 'Color', c_blue, ...
                 'FontWeight','bold','HorizontalAlignment','center','FontSize', fs);
        end


        % Row labels (right-most column): baseline schedule labels (FIXED)
        if j == numel(schedules)
            xl_row = xlim; yl_row = ylim;
            x_pos  = xl_row(2) * 1.02;                 % just outside right edge
            y_pos  = mean(yl_row);
            text(x_pos, y_pos, base_sched, 'Color', c_red, ...
                 'FontWeight','bold','FontSize', fs, ...
                 'HorizontalAlignment','left', 'VerticalAlignment','middle');
        end


        % Background tint by estimated AI volume
        est_vol_mL = dose_match_mg / formulation_mg_mL;
        if est_vol_mL <= ai_bins_mL(end)
            kbin = find(est_vol_mL <= ai_bins_mL, 1, 'first');
            set(gca, 'Color', ai_bin_colors(kbin,:));
        else
            set(gca, 'Color', overflow_color);
```

```
        end


        box on; hold off;
    end
end


% Global labels
xlabel(tl, 'Time (weeks)', 'FontWeight','bold','FontSize', fs);
ylabel(tl, 'Concentration (nM)', 'FontWeight','bold','FontSize', fs);
title(tl, sprintf('Dose-Matching by C_{avg,ss} to %g mg %s (rows) vs. SC + rHuPH20 (columns)',
...
      baseline_dose_mg, baseline_route), ...
      'FontWeight','bold','FontSize', fs+1);


%% ============================ FUNCTIONS ============================ %%

function [t, y] = runPkModel(p, cfg)
%RUNPKMODEL  Two-compartment PK with first-order SC absorption.
%
% Inputs
%   p   : struct with fields {Vc,Vp,CL,Q,ka,F,MW,kel,k12,k21}
%   cfg : struct with fields {route,schedule,dose_mg,t_meas_days}
%
% Outputs
%   t   : time vector (days)
%   y   : [A_sc (nmol), Cc (nM), Cp (nM)]


    % Initialize state and time
    t = 0;
    y = zeros(1,3);   % [A_sc, Cc, Cp]


    % Build dose times (days), plus terminal point to close last interval
    t_dose = [scheduleToTimes(cfg.schedule, cfg.t_meas_days(end)), max(cfg.t_meas_days)];


    % Integrate between dosing times with bolus at left boundary
    for k = 1:(numel(t_dose)-1)
        t1  = t_dose(k);
        t2  = t_dose(k+1);
        y0  = y(end,:);


        switch upper(cfg.route)
            case 'SC'
                % mg -> nmol: 1e6 / MW ; apply F; add to SC depot
```

```
                y0(1) = y0(1) + cfg.dose_mg * p.F * (1e6/p.MW);
            case 'IV'
                % mg -> nmol then to nM by dividing by Vc (L)
                y0(2) = y0(2) + (cfg.dose_mg * (1e6/p.MW)) / p.Vc;
            otherwise
                error('Unsupported route: %s (use ''SC'' or ''IV'')', cfg.route);
        end


        % Solve ODEs from t1 to t2 using daily steps (fits the measurement grid)
        [Tseg, Yseg] = ode15s(@(tt, yy) pkOde(tt, yy, p), t1:1:t2, y0);
        t = [t; Tseg];
        y = [y; Yseg];
    end


    % Remove duplicated initial row
    t(1,:) = [];
    y(1,:) = [];
end


function dydt = pkOde(~, y, p)
%PKODE  Two-compartment linear PK with first-order SC absorption.
% y(1) = A_sc (nmol), y(2) = Cc (nM), y(3) = Cp (nM)


    A_sc = y(1);
    Cc   = y(2);
    Cp   = y(3);


    dydt    = zeros(3,1);
    dydt(1) = -p.ka * A_sc;
    dydt(2) =  p.ka*A_sc/p.Vc - p.k12*Cc + p.k21*Cp*(p.Vp/p.Vc) - p.kel*Cc;
    dydt(3) =  p.k12*Cc*(p.Vc/p.Vp) - p.k21*Cp;
end


function t_dose = scheduleToTimes(schedule, t_final_days)
%SCHEDULETOTIMES  Map schedule string to dose times (days).
% Only schedules used in this figure are provided to keep the supplement concise.


    if nargin < 2, t_final_days = 0; end


    switch upper(schedule)
        case {'X1'}
            t_dose = 0;
```

```
        case {'QD'}
            t_dose = 0:1:t_final_days;      t_dose(end) = [];
        case {'QW'}
            t_dose = 0:7:t_final_days;      t_dose(end) = [];
        case {'Q2W'}
            t_dose = 0:14:t_final_days;     t_dose(end) = [];
        case {'Q4W','QM'}
            t_dose = 0:28:t_final_days;     t_dose(end) = [];
        case {'Q8W','Q2M'}
            t_dose = 0:56:t_final_days;     t_dose(end) = [];
        case {'Q12W','Q3M'}
            t_dose = 0:84:t_final_days;     t_dose(end) = [];
        case {'Q24W','Q6M'}
            t_dose = 0:168:t_final_days;    t_dose(end) = [];
        otherwise
            error('Unrecognized schedule: %s', schedule);
    end
end
```
